# Design and implementation of a cross-sectional nutritional phenotyping study in healthy US adults

**DOI:** 10.1186/s40795-017-0197-4

**Published:** 2017-10-19

**Authors:** Lacey M. Baldiviez, Nancy L. Keim, Kevin D. Laugero, Daniel H. Hwang, Liping Huang, Leslie R. Woodhouse, Dustin J. Burnett, Melissa S. Zerofsky, Ellen L. Bonnel, Lindsay H. Allen, John W. Newman, Charles B. Stephensen

**Affiliations:** 1 United States Department of Agriculture, Agricultural Research Service, Western Human Nutrition Research Center, Davis, CA USA; 2 Department of Nutrition, University of California Davis, Davis, CA USA; 3 NIH West Coast Metabolomics Center, Davis, CA USA

**Keywords:** Diet, Metabolism, Phenotype, Metabotypes, Gut microbiota, Metabolomics, Neuroendocrine system

## Abstract

**Background:**

Metabolic imbalance is a key determinant of risk of chronic diseases. Metabolic health cannot be assessed solely by body mass calculations or by static, fasted state biochemical readouts. Although previous studies have described temporal responses to dietary challenges, these studies fail to assess the environmental factors associated with certain metabolic phenotypes and therefore, provide little scientific rationale for potentially effective intervention strategies.

**Methods/design:**

In this phenotyping study of healthy US adults, we are evaluating lifestyle, biological and environmental factors in addition to metabolic parameters to determine the factors associated with variations in metabolic health. A series of practical fitness, dietary, and emotional challenges are introduced and temporal responses in various areas of specialization, including immunology, metabolomics, and endocrinology, are monitored. We expect that this study will identify key factors related to healthy or unhealthy metabolic phenotypes (metabotypes) that may be modifiable targets for the prevention of chronic diseases in an individual.

**Discussion:**

This study will provide novel insights into metabolic variability among healthy adults in balanced strata defined by sex, age and body mass index. Usual dietary intake and physical activity will be evaluated across these strata to determine how diet is associated with health status defined using many indicators including immune function, metabolism, body composition, physiology, response to exercise andmeal challenges and neuroendocrine assessment. A principal study goal is to identify dietary and other personal factors that will differentiate different levels of "health" among study participants.

**Trial registration:**

ClinicalTrials.gov NCT02367287.

## Background

Current nutrition recommendations in the US are for all Americans regardless of key characteristics that distinguish individuals from one another. Although the dietary guidance is practical and provides a link between scientific research and the US consumer, it fails to address the multitude of physiological, behavioral, and environmental factors that may affect the nutritional needs of an individual. Many biological factors have been identified as contributors to health and risk of disease. For example, the composition of the gut microbiota may have a causal role in the development of obesity [1] and in the pathogenesis of obesity-related metabolic diseases [1, 2]. Immune activation and inflammation have been demonstrated, along with elevated blood triglycerides, to respond to a high-fat meal, suggesting a pro-inflammatory effect of a dietary fat challenge [3]. Diet-induced changes in endocrine function are also thought to impact metabolic health. Elevated serum leptin and low adiponectin concentrations are recognized as obesity comorbidities that may establish a postprandial pro-atherogenic state [4] and dietary fats can modulate appetite by decreasing intestinal levels of anorectic lipids [5].

Previous phenotyping studies have identified metabolic health as a differentiating factor among individuals with normal, overweight, or obese body sizes. Regardless of body size, a “metabolically healthy” individual refers to a person who responds to a high-fat dietary challenge with a greater degree of phenotypic flexibility (i.e. the capacity to maintain or regain homeostasis in response to an acute dietary challenge) thus proving more adaptable to alterations in external factors [6]. In this example, metabolic health was defined using static measurements taken during the fasted state that reflect cardiometabolic risk factors (e.g. hypertension, insulin resistance, decreased HDL cholesterol levels, etc.) [7]. While body size may be an indicator of metabolic abnormality risk, findings suggest that additional factors may explain more variations in phenotypic flexibility across populations.

As an example, habitual dietary intake may correlate with phenotypic flexibility. It represents a chronic metabolic challenge that may negatively or positively influence phenotypic flexibility. Kardinaal et al. found that healthy individuals given a 4 week high-fat and high-caloric diet intervention exhibited changes in adipose tissue mass and function, metabolic flexibility (the capacity to switch between glucose and fat as fuel), vascular health, and glucose metabolism compared to the metabolic parameters collected before the high-fat and high-caloric intervention [8]. Fasting total cholesterol levels and several appetitive and metabolism-related hormones were altered in healthy subjects after consuming the high-fat and high-caloric diet, which greatly resemble the characteristics of individuals in the metabolic syndrome group. In addition, differential responses to a high-fat challenge meal were observed in healthy subjects after the high-fat and high-caloric intervention, including postprandial biomarkers of systemic stress and inflammation, metabolic adaptation, vascular health, and glucose metabolism [8].

As the obesity epidemic with its associated increased risk of chronic disease broadens in the US and globally, it is apparent that novel preventative strategies are needed. We believe that population stratification (i.e. phenotyping) by metabolic response will allow new investigations to assess metabolic risks of lifestyle including habitual diet and exercise patterns and to determine how malleable metabolic responses are within and among each strata.

This study aims to expand the parameters used to define phenotypes by observing temporal changes in immunologic, physiologic, and metabolomic endpoints in response to a standard dietary challenge. Furthermore, a comprehensive panel of assessments and observations will be used to identify the correlates of these phenotypic profiles. Identification of factors or genes related to physiology, behavior, or the environment may serve as targets for future interventions that aim to maximize phenotypic flexibility. The purpose of this paper is to describe the study design in terms of a priori hypotheses to be tested, study populations, data collection methods, laboratory methods, and general statistical analysis plans.

### Study questions

1. Are there key lifestyle and/or physiological factors that differentiate generally healthy individuals in the US population in terms of phenotypic flexibility and are these major sources of variation associated with inflammatory, metabolic, and other physiologic responses to a dietary challenge?
2. Is there a relationship between diet quality and the temporal sequence of systemic immune activation and inflammation in response to a mixed macronutrient challenge meal?
3. Is there an association between diet quality and the temporal sequence of the systemic metabolome in response to a mixed macronutrient challenge meal?

### Primary hypotheses

1. We hypothesize that diet quality, eating behavior, physical fitness, gut microbiota, and allostatic load will be the major factors correlated with phenotypic flexibility, especially the temporal sequences of systemic immune activation and inflammation and the systemic metabolome in response to a dietary challenge.
2. We hypothesize that higher Healthy Eating Index scores, and other measures of diet quality, will be associated with lower risk of dysbiosis of the gut microbiota and in turn, lower gut inflammation. These factors will also be associated with lower systemic immune activation and inflammation in response to a mixed macronutrient challenge meal.
3. We hypothesize that higher diet quality will be associated with lower postprandial fatty acid concentrations and other markers of inflammation (plasma metabolomics), and reduced evidence of hepatic de novo lipogenesis in response to a mixed macronutrient challenge meal.

### Secondary hypotheses

Secondary endpoints are grouped into three categories: metabolic/physiologic impacts, neurocognitive function, and diet/lifestyle/personal characteristics. Each category is defined below according to the list of dependent variables to be included. Selected secondary a priori hypotheses are numbered following each category definition and identified by the independent variable of interest. Directionality of the hypothesized association may vary according to each independent/dependent variable combination and therefore, the expected direction of the independent variable is reported in the numbered section and the expected direction of the dependent variable is reported in the category definition (e.g. higher diet quality will be associated with higher insulin sensitivity and lower glucose concentration), see below.

The metabolic/physiologic category includes: higher insulin sensitivity, substrate switching, production of short-chain fatty acids, breath hydrogen and methane, subjective satiety, and pulmonary function; and lower glucose concentration, peripheral arterial tone, risk of dysbiosis of the gut microbiota, gut inflammation, systemic immune activation, systemic inflammation, fatty acid profiles, oxygenated metabolites, endocannabinoids, bile acid profiles, hepatic lipogenesis, triglyceridemia, appetitive hormones, and taste threshold.

1. Higher diet quality (e.g. according to the Healthy Eating Index), modified by genotype, will be associated with metabolic/physiologic phenotypes.
2. Healthy eating behaviors (health-related food choices motives and non-addictive eating behaviors) will be associated with metabolic/physiologic phenotypes.
3. Dysbiosis and gut inflammation will be associated with metabolic/physiologic phenotypes.
4. Lower postprandial bile acid responses will be associated with metabolic/physiologic phenotypes.
5. Lower postprandial plasma fatty acid concentrations will be associated with metabolic/physiologic phenotypes.

The neurocognitive function category includes higher executive function, healthy eating behaviors, and stress system responsiveness, and lower chronic stress, allostatic load, heart rate variability, respiration variability, and nerve conductivity.

## Higher physical activity will be associated with neurocognitive function and metabolic/physiologic phenotypes

The diet/lifestyle/personal characteristics category includes demographics, higher diet quality, nutrient status, physical activity, bone mineral density, health-related food motives, and lower body fat, waist: hip ratio, and body mass index.

6. Lower stress and allostatic load will be associated with diet/lifestyle/personal characteristics, neurocognitive function, and metabolic/physiologic phenotypes.
7. Higher executive function will be correlated with diet/lifestyle/personal characteristics and neurocognitive function.

## Methods/design

### Study approvals

The study is registered on *ClincialTrials.gov* (Identifier: NCT02367287) and received ethical approval from the University of California Davis Institutional Review Board.

### Participant population

This study is being carried out at the United States Department of Agriculture, Agriculture Research Service Western Human Nutrition Research Center located on the University of California Davis campus in Davis, CA. Beginning in May of 2015, generally healthy people living near Davis, CA were being invited to participate in this cross-sectional study involving a two-week observational period. It is anticipated that recruitment will continue for four years. Interested volunteers are invited to complete a screening interview if they are 18–65 years of age, male or female, and have a body mass index (BMI) 18.5–39.9 kg/m^2^ (normal to moderately obese). Individuals are excluded if they are pregnant or lactating women, have a known allergy to egg, have recently undergone a minor surgery, recently received antibiotic therapy, or have been hospitalized within 4 wk. Volunteers are excluded if they have had a major surgery within the past 16 wk, are currently taking daily medication for a diagnosed chronic diseases including but not limited to: diabetes mellitus, cardiovascular disease, cancer, gastrointestinal disorders, kidney disease, liver disease, bleeding disorders, asthma, autoimmune disorders, hypertension, and osteoporosis, or are using prescription medications at the time of the study that directly affect study endpoints of interest (e.g. hyperlipidemia, glycemic control, steroids, statins, anti-inflammatory agents, and over-the-counter weight loss aids). In addition, the screening interview includes an open-ended question about food allergies, intolerances or sensitivities and test-specific questions for exclusion from the YMCA step test. The presence of a pacemaker or similar device or moderate knee or hip pain will exclude an individual from the YMCA step test, but not from the study.

A sampling scheme consisting of 18 categories defined by age, sex, and BMI are designed to be filled at a relatively even rate to balance enrollment across seasons and years. The categories have been defined according to predetermined participant characteristics (see study inclusion criteria). There are nine sampling bins for each sex, male and female; there are three BMI categories within each of the three age categories (Table 1). The age range is divided into three categories each comprised of 16 years (18.00–33.99 y, 34.00–49.99 y, 50.00–65.00 y) and the BMI range is divided into three categories according to WHO international classifications (normal: 18.50–24.99 kg/m^2^, overweight: 25.00–29.99 kg/m^2^, and obese 30.00–39.99 kg/m^2^). The study year is divided into trimesters with a target enrollment of two and no more than three participants enrolled in each sampling bin per trimester. Target enrollment for each of four study years is 109 based on facility and resource use capacity limitations.

**Table 1:** Participant sampling scheme. Participants are categorized into 18 bins according to sex, age, and BMI

Sex	Age (y)	BMI	Sampling Bin
Male	18–33	<25.0	1
		25–29.9	2
		30–39.9	3
	34–49	<25.0	4
		25–29.9	5
		30–39.9	6
	50–65	<25.0	7
		25–29.9	8
		30–39.9	9
Female	18–33	<25.0	10
		25–29.9	11
		30–39.9	12
	34–49	<25.0	13
		25–29.9	14
		30–39.9	15
	50–65	<25.0	16
		25–29.9	17
		30–39.9	18

Volunteers are being recruited using email lists, flyers, newspaper advertisements, radio spots, mailers, and study staff presence at local fairs. Data reports of body composition and bone mineral density (dual energy x-ray absorptiometry), nutrient intake (food frequency questionnaire), resting metabolic rate (indirect calorimetry) and energy expenditure (accelerometer) are provided to study participants when available and participants receive up to $150 for completing the protocol and return of study equipment.

### Study design overview

This study is designed as a cross-sectional observational trial requiring two study visits within a period of 10–14 d. Interested volunteers contact the study coordinators by phone or email. The initial screening interview assesses sex, age, and estimated BMI in order to determine the sampling bin and collects recent medical history to address the inclusion and exclusion criteria. Volunteers are advised to postpone enrollment in the study if blood has been donated in the past 8 weeks.

Testing does not commence until informed consent is obtained in-person during the first visit (Visit #1). A secondary screening of vital signs may exclude individuals with elevated blood pressure or body temperature (active infection). Anthropometry, body composition and physiology assessments are performed and training for at-home dietary data collection, at-home biological specimen collection, and preparation instructions for the full-day visit (Visit #2) are provided. Training includes instructions and demonstration of proper placement of an accelerometer, self-administered 24-h recall, urine and stool collections, and how to follow the study foods consumption protocol. A standard meal is provided during Visit #1 for consumption the evening before Visit #2. After the meal is consumed, participants fast for 12 h overnight, maintaining normal water consumption. Concurrently, participants collect a 12-h overnight urine sample and a single stool specimen as close to Visit #2 as possible.

Participants are asked to refrain from taking over-the-counter medications and nutritional or herbal supplements for a 3-d washout period and to avoid strenuous physical activity 24 h before Visit #2. Biological specimens, pretest meal supplies, and the accelerometer are returned at the start of Visit #2. The test day commences with assessments of vital signs, anthropometrics, symptoms-based interview, and a recent history of medication use (3 d). Elevated blood pressure or body temperature and recent upper respiratory or gastrointestinal symptoms warrant Visit #2 rescheduling.

**Fig. 1:**
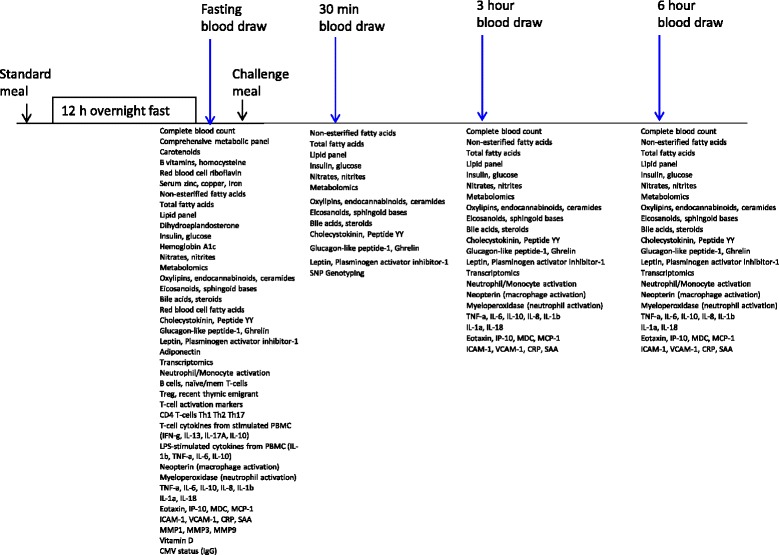
Timeline of blood sampling and planned assays by sample collection time point in relation to challenge meal

Resting metabolic rate is assessed first, following the 12-h fast. Fasting samples of biological specimens are collected (breath, saliva, and blood) and baseline assessments of subjective hunger and appetite are performed before the dietary challenge. The participant is asked to consume the mixed macronutrient challenge meal within a 10 min period and timed collections of each specimen or assessment are calculated from the challenge end time (timing details provided below). Before the dietary challenge, electrodes are placed on the body for autonomic nervous system monitoring (MindWare technology). Salivary cortisol samples are collected at 30 min intervals following the challenge, up to a period of 90 min. Blood samples are collected at 30 min, 3 h, and 6 h after the challenge meal (Fig. 1).

The food preference activity and food frequency questionnaires are conducted approximately 1.5–2 h after the meal. Upon completion of the saliva collection period, an emotional challenge is introduced by asking the participant to recall an unresolved conflict that is still a source of anger. Heart rate variability, skin conductance, and salivary cortisol are monitored over the course of the emotional challenge and post-emotional challenge rest periods and activities. Between the timed biological specimen collections, several questionnaires are administered using an electronic survey format. Skin reflectance measurements using spectrophotometry are also performed. Unscheduled time during the visit provides the participant several periods of rest. The afternoon snack is provided after the final blood draw and a cognitive assessment is performed immediately after. An endothelial function assessment and taste threshold tests are the final assessments.

Follow-up is required only in the event that study equipment is not returned, a specimen is not provided, or the at-home dietary data collection is not completed.

## Data collection protocols

### Questionnaires

Questionnaires are administered by electronic survey during both visits using tablets, laptops, or a desktop computer. The demographic questionnaire was developed specifically for this study and includes current city, birthplace, race or ethnicity, tobacco use, sleep hours, sun exposure, household characteristics, and education, employment, and income information. Other questionnaires being administered include Stanford Brief Physical Activity Survey [9], Yale Food Addiction Scale [10], Profile of Mood States© [11], Food Choice Questionnaire [12], Three-Factor Eating Questionnaire (Eating Inventory©) [13], and Wheaton Chronic Stress Inventory [14]. Licenses for electronic administration of questionnaires were obtained for all copyrighted materials.

The survey feature of the REDCap™ database [15] is used to display instructions, questions, and response options exactly as they appear on the validated tools. Each survey entered has a unique URL self-administration, allowing respondent selections to be directly and confidentially entered into the study database. Data quality assurance checks notify respondents if a survey question is left blank or if multiple selections are made for a single question. Responses are later downloaded from the database and scored according to published scoring instructions.

### Vital signs, anthropometry and body composition

Blood pressure, pulse, and body temperature are evaluated using GE® DINAMAP™ vitals monitors at the start of each study visit. All excess clothing (shoes, hats, jackets, scarves, etc.) and equipment (phones, keys, backpacks, etc.) are removed and height and weight are measured in duplicate to the nearest 0.1 cm and 0.1 kg, respectively. Measurements are repeated a third time if the repeated measurements differential is greater than 0.2 kg or 0.2 cm. Permanently mounted stadiometers are used to assess height and electronic scales are used to assess weight (Scale-tronix 6002 or Tanita® BWB-627A). BMI is calculated from the anthropometric measurements to verify the sampling bin classification. Waist and hip circumference are measured by trained study personnel using a non-elastic tape measure. Waist circumference is measured at the smallest horizontal circumference between the ribs and iliac crest. Hip circumference is measured at the greatest extension of the posterior.

Body composition and bone mineral density are reported overall and by body region. All female participants are asked to report the date of their last menstrual period and to complete a spot urine pregnancy test, except in cases of reported hysterectomy or self-reported menopause (> one year since last menstrual period). A whole body and two-site specific DXA scans are performed by CA licensed Limited Use X-ray Technicians using Hologic® Discovery™ QDR® Series 84,994 instrumentation. The whole body scan captures total lean mass, total fat mass, and percentages of lean and fat mass. Bone mineral content and density are calculated based on two site-specific scans: 1) neck of the left femur and 2) lumbar spine.

### Physiological data collection

#### Fitness

The YMCA step test is a submaximal physical fitness challenge performed during Visit #1. Participant responses to the Physical Activity Readiness Questionnaire (American College of Sports Medicine) are reviewed by a physiologist as precautionary screening prior to administration of the fitness challenge. A Polar® V800 heart rate monitor is placed directly on the skin near the sternum and secured in place with an appropriately-sized band. The participant is asked to step up and down on a 12 in. box for three min. The stepping rate is coordinated to the beat of a metronome, set to 24 step-ups per min and the task is demonstrated to the participant before beginning.

#### Physical activity energy expenditure

Energy expenditure from physical activity is monitored for a period of 7 d between the study visits using a Respironics® Actical™ accelerometer. Participants record sleeping and waking times, times that the device was removed and reason for removal (e.g. due to water exposure during swimming, bathing, etc.), additional activities performed and instances of discomfort, etc. on an accelerometer use log. The accelerometers are pre-programmed with sex, age, and anthropometric data for each respective participant and a fitted belt is provided for secure placement over the left hip. Time spent performing physical activity at sedentary, light, moderate and vigorous activity levels (using the default activity versus time settings supplied by the manufacturer) are recorded and energy expenditure in kilocalories is calculated for respective activity levels. Actical™ data is extracted using an ActiReader™. Sedentary activity is also observed by the device and sleep times are estimated according to recorded times on the accelerometer use log report.

#### Respiratory function

Standard measures of spirometry are used to assess breathing and pulmonary function using the SDI Diagnostics Spirolab II ver. 3.4 or AstraTouch Spirometer. Participants are asked to inhale a full, deep breath and exhale as forcefully as possible using a single use mouthpiece. The test is administered 3 times and the best score is recorded. Forced vital capacity is used to measure the total volume of air exhaled in a forced expiratory maneuver (the act of exhaling as hard and fast as possible after maximal inspiration). The forced vital capacity is used for detection of obstructive or restrictive diseases. The forced expiratory volume at one second is the amount of air that a person exhales during the first second of a forced expiratory maneuver and is expressed as a percentage of the predicted volume. Forced expiratory volume at one second /forced vital capacity is the most sensitive and specific index of airway obstruction measured by spirometry.

#### Metabolic rate

The metabolic rate is assessed by trained physiologists using indirect calorimetry. Automated metabolic carts (TrueOne 2400, Parvo Medics) measure resting and postprandial metabolic rates using an open circuit system. Measurement times coincide with blood collection times: 0 h (fasting), 45 min-1 h, 3 h, and 6 h postprandial. Participants are asked to rest quietly for 5–10 min before beginning the assessment, and collection times are approximately 15–20 min. Respiratory gases are collected while participants are in a semi-reclined position, wearing a facemask that fits securely, covering the nose and mouth. The facemask is attached to a tubing assembly connected to the cart’s mixing chamber. Through the facemask, participants breathe room air, and their exhalations are trapped by the mask and directed to the mixing chamber for volume and gas analyses. Respiratory exchange ratio is calculated using observed oxygen consumption (VO_2_) and carbon dioxide (VCO_2_) production. The Weir equation with and without urinary nitrogen is also used to estimate metabolic rate. Urinary nitrogen excretion rate is estimated using reported nitrogen intake from the 24-h dietary recalls and assuming that participants are in nitrogen balance. Frayn equations for fat and carbohydrate oxidation are used to estimate substrate utilization using VO_2_ and VCO_2_ and urinary nitrogen as described above.

### Dietary intake assessments

Two dietary assessment approaches are being used in this study. First, recent intake is assessed using the ASA24™ automated self-administered 24-h recall from the National Cancer Institute of the National Institutes of Health [16]. Measuring cups and spoons are presented on-site to demonstrate serving sizes. Participants are sent unscheduled email prompts to complete self-administered 24-h dietary recalls on two weekdays and one weekend day between the scheduled study visits (approximately 10–14 d). The recall period is from midnight to midnight the day prior to receipt of the prompt. In the event that participants do not have computer or internet access, the participant is invited to complete the 24-h recall using an on-site computer.

Habitual dietary intake is assessed using the Block 2014 Food Frequency Questionnaire by NutritionQuest [17]. Estimates of frequency of consumption of certain foods over the past 12 months are used to avoid potential seasonal biases of food intake. The electronic version of the questionnaire is administered by interview with trained study personnel and measuring cups and spoons are present for estimation of serving sizes.

### Study foods

A standard pre-test meal is provided to participants for consumption between 18:00 and 19:00 the evening before the scheduled visit #2. The targets for macronutrient intake include 30% kcal from fat, 55% kcal from carbohydrates, and 15% kcal from protein. All study foods were designed by a registered dietitian, calculated using the Nutrient Data System for Research version 2014, are prepared by the WHNRC Metabolic Kitchen and Human Feeding Laboratory, are ovo-vegetarian, and omit milk, peanuts, tree nuts, finfish, shellfish, wheat or soy as potential allergens. Written food handling and safety instructions and retherming instructions are provided by the study dietitian and included with the frozen foods. Participants are shown how to record the approximate time of consumption and estimate percent consumption of each of the study foods using a provided meal checklist. Participants are instructed to return all food packaging and uneaten foods for determination of plate waste. Detailed timing, amount, and descriptions of any additional foods, drinks, supplements, or medications consumed after 19:00 are also recorded on the meal checklist. An estimate of the time of the last meal consumed prior to the pre-test meal is also recorded. Participants are also encouraged to report any symptom-based complaints related to study foods, including but not limited to gas, bloating, diarrhea, headache, or nausea.

### Mixed macronutrient meal challenge

A mixed macronutrient liquid challenge meal patterned after a similar meal developed by the European Nutrigenomics Organization [18] contains the main ingredients: palm oil, sucrose, and pasteurized liquid egg white. Xanthan gum and cellulose gum standardized with maltodextrin are added as emulsifying agents; vanilla and almond extracts and artificial butter flavoring are added to improve palatability. All participants receive a standard meal volume, which totals approximately 12 fluid oz. The target caloric load is 800 kcal, consisting of approximately 60% kcal from fat, 25% kcal from carbohydrates, and 15% kcal from protein. Baseline assessments of vital signs, resting metabolic rate, gut fermentation (breath sample), cortisol (saliva sample), blood specimens, subjective feelings of hunger and appetite, and MindWare data collection (see description below) are completed in the fasting state before the challenge meal is provided. A small volume of deionized water (60.0 mL) is provided with the challenge meal. Additional water intake is restricted until after the 0.5 h postprandial blood draw. Deionized water is then provided for ad libitum consumption throughout the remainder of the day.

A standard snack is provided after the 6 h postprandial blood draw. The snack contains limited plant-derived polyphenols or flavonoids due to the effects of these compounds on vasodilation. Similarly, it is ovo-vegetarian and does not include the potential allergens previously listed.

### Biological specimens

#### Urine

Urine is collected for 12 h overnight prior to visit #2. Participants are instructed to void the first urine at 18:00, the time that the pre-test meal is consumed. All subsequent urine is collected in a weighed 24-h urine collection jug and stored in a provided cooler on blue ice. The final urine collection occurs at 06:00, before traveling to the research center. Urine weight is recorded and processing occurs the same day. Urine is thoroughly mixed and untreated urine is stored in 2 mL and 1 mL aliquot volumes. Acidification of 10 mL urine to 0.03 M HCl is performed using 6 M HCl (Fisher Trace Element Grade) and stored in 1 mL aliquot volumes. All specimens are stored in Cryo-Store® vials at -80 °C.

#### Stool

A single stool specimen is collected within 3 d of the scheduled visit #2. Sanitary collection supplies are provided and participants receive an email reminder 3–4 d prior to the visit. The stool is kept on blue ice and transported to the research center as soon as possible, for same-day processing. The specimen is homogenized using a Stomacher® paddle blender. The specimen is frozen and scored using sharp instruments for division into Cryo-Store® vials for long-term storage at -80 °C.

#### Saliva

Saliva is collected using the SalivaBio Oral Swab method by Salimetrics®. Saliva is collected immediately before and at 30 min intervals, up to 90 min, following each of three challenges: 1) physical fitness, 2) dietary, and 3) emotional. Sterile swabs are placed in the mouth and chewed for two min. Filled swabs are returned to the salivette container, sealed, and stored at room temperature until all daily samples are collected. Samples are transferred to 4 °C refrigeration for approximately one wk, until processing. Saliva is extracted from the collection swabs by centrifugation at 1500 × g, 21 °C, for 15 min and transferred to -80 °C storage until analysis.

#### Breath

Breath samples are collected hourly throughout the research day. Participants are instructed to inhale normally and fully exhale, in an effort to completely evacuate the lungs. Breath is collected using a single patient QuinTron© collection system and analyzed in real-time for carbon dioxide, hydrogen, and methane concentrations using the QuinTron© BreathTracker™ instrumentation.

#### Blood

Certified phlebotomists collect blood by venipuncture at four time points during Visit #2: 1) fasting, 2) 0.5 h postprandial, 3) 3 h postprandial, and 4) 6 h postprandial. All blood specimens remain on wet ice for 30 min before processing, except for the following, which require room temperature: serum, Na Heparin, and K_2_ EDTA plasma for complete blood count. Serum and plasma vacutainers are centrifuged at 1300 x g at 4 °C for 10 min. Aliquots are prepared immediately and stored in Cryo-Store® vials at -80 °C until analysis.

## Laboratory methods

### Assessment of adaptive and innate immune function

Relative frequencies of specific cell types are obtained by flow cytometry (BD LSRFortessa™). Peripheral blood mononuclear cells (PBMC) are isolated from whole blood buffy coats by Ficoll density gradient centrifugation. Lymphocyte subsets are characterized based on expression of surface proteins and include: memory and naïve B cells, memory and naïve CD4+ and CD8+ T cells, regulatory T cells, type-1 helper T cells (Th1), type-2 helper T cells (Th2), type-17 helper T cells (Th17). Innate immune cells (monocytes, neutrophils, eosinophils) are enumerated in whole blood and identified by flow cytometry. T cell responses are measured in freshly isolated peripheral blood mononuclear cells cultured with anti-CD3 and anti-CD28 antibodies or isotype control antibodies for 48 h to stimulate production of Th1 (IFN-γ), Th2 (IL-13), Th17 (IL-17A) and regulatory (IL-10) T cell cytokines. Innate immune responses are measured in freshly isolated PBMC cultured with bacterial lipopolysaccharide (LPS) for 24 h to stimulate monocyte cytokine (IL-1β, TNF-α, IL-6, IL-10) production. The concentrations of cytokines in culture supernatants are quantified by electrochemiluminescent multiplex assays [Meso Scale Discovery Sector Imager 2400].

Plasma markers of inflammation including acute phase proteins (C-reactive protein and serum amyloid A), markers of vascular inflammation (ICAM-1 and VCAM-1), chemokines (eotaxin, IP-10, MDC, MCP-1), cytokines (TNF-α, IL-6, IL-10, IL-8, IL-1β), and markers of innate immune activation (neopterin and myeloperoxidase) are measured by ELISA or electrochemiluminescent multiplex assays [Meso Scale Discovery Sector Imager 2400].

### Identification of intestinal microbiota and inflammation

The relative abundances of stool bacteria are characterized by sequence analysis of the 16S ribosomal RNA genes. Microbiome analysis of raw DNA sequencing data is performed using Quantitative Insights into Microbial Ecology bioinformatics software. Neopterin and myeloperoxidase in stool samples will be measured by ELISA.

### Metabolomics

Metabolomic profiles are determined using both quantitative targeted and semi-quantitative untargeted mass spectrometric methods. Endpoints include but are not limited to non-esterified fatty acids, total fatty acids and oxylipins, endocannabinoids, NS-ceramides, sphingoid bases, bile acids, steroids, acylcarnitines, amino acids, biogenic amines, phospholipids, triglycerides, sphingomyelins, and cholesteryl esters. Prior to analyses, samples are enriched with isotopically labeled non-native analytes to be used as analytical surrogates, and samples are randomized across analytical batches, each containing a laboratory reference material to document analytical stability. Samples are randomly replicated at a rate of 5% across the study.

### Chronic stress and stress reactivity

Allostatic load is an aggregate value derived from several parameters reflecting stress exposure. Concentrations of cortisol, epinephrine, norepinephrine, and creatinine in a 12-h urine sample are determined by ELISA. Hemoglobin A1c is measured in whole blood using the Cobas® Integra® 400 plus. Dehydroepiandosterone-sulfate concentrations are determined in plasma by electrochemiluminescence using a Cobas® e411 Immunology analyzer. Other parameters included in the allostatic load score were previously described (resting blood pressure, waist: hip circumference ratio, cholesterol level, and high sensitivity C-reactive protein). Acute stress system reactivity is measured by examining autonomic nervous system and endocrine responses to the meal, physical fitness, and emotional challenge tasks.

### Endocrine function

Endocrine hormone concentrations (cholecystokinin and peptide YY) are quantified by radioimmunoassay and gamma counter (Packard Cobra II Auto Gamma), Meso Scale Discovery multiplex panels (ghrelin, glucagon-like peptide-1, plasminogen activator inhibitor-1, adiponectin and leptin) and with the Cobas® e411 instrument (insulin). Blood specimens are collected in vacutainers containing EDTA plus protease inhibitor additives: DPPIV inhibitor (dipeptidyl-peptidase IV inhibitor (Millipore, St. Charles, MO, USA) 10 μl/ml whole blood) and aprotinin (G-Biosciences, St. Louis, MO, USA) 240 KIU/ml whole blood). In addition, for the ghrelin aliquot, a 1 M HCl (Trace Metal Grade HCl, Fisher-Scientific, Pittsburg, PA, USA) solution is added (100 μl/ml plasma) prior to plasma storage at −80C.

Plasma glucose and lipid concentrations and serum non-esterified fatty acid concentrations are measured using the Cobas® Integra® 400 plus.

### Genomics and transcriptomics

Genomic DNA is isolated from whole blood (8 mL) using Qiagen© PAXgene Blood DNA kits and purified genomic DNA is aliquoted and stored at -80 °C until use. Single nucleotide polymorphisms in the genes of interest will be analyzed using TaqMan® SNP Genotyping Assays (ThermoFisher Scientific) performed on a QuantStudio® 7 Flex Real-Time PCR System (ThermoFisher Scientific) according to the manufacturer’s instructions. Allelic discriminations will be performed using Genotyper™ software (ThermoFisher Scientific). All ambiguous genotypes will be repeated in independent PCR experiments.

Transcriptomic profiling of whole blood is planned for a subset of participants to assess systemic immune activation. The Tempus™ blood RNA collection tube and the Tempus™ Spin RNA isolation kit (ThermoFisher Scientific) is used to stabilize and to isolate RNA.

### Nutrient status

Plasma carotenoid levels are quantified by liquid chromatography-diode array detection. Plasma homocysteine, thiamin, vitamin B6 and red blood cell riboflavin levels are quantified by liquid chromatography-fluorescence detection. Plasma vitamin B12 and folate status are analyzed by electrochemiluminescence immunoassay on Cobas®e411 analyzer.

Concentrations of trace minerals including iron, zinc, and copper are determined by Inductively Coupled Plasma-Optical Emission Spectrophotometry (Vista, Agilent Technologies). Blood is collected using Sarstedt Lithium Heparin polypropylene tubes.

### Clinical chemistry

Specimens required for clinical laboratory panels are submitted to the University of California Davis Medical Center clinical laboratory following the specified collection requirements. Comprehensive metabolic panels are determined by automated chemical analyzer. Complete blood count with differential analysis is quantified by Coulter count.

### Cognitive testing

The Wechsler Abbreviated Scale of Intelligence™ Vocabulary and Matrix Reasoning modules are administered to assess cognitive function. A trained interviewer leads the assessments and records the responses given without providing indication of performance. The WASI®-II Stimulus Book is placed directly in front of the respondent to provide visual prompts for each task. The interviewer provides a standard set of instructions and begins when the participant is ready. Responses are scored by the interviewer in real-time according to published instructions for both modules. The T score, intelligence quotient, and 95% confidence interval are calculated according to published instructions.

The Cambridge Neuropsychological Test Automated Battery® is administered to assess cognitive function, including executive function. CANTAB® eclipse™ 5 software by Cambridge Cognition Ltd. is licensed on a touch screen tablet computer to complete each of the following four modules: 1) Motor Screening, 2) One Touch Stockings of Cambridge, 3) Spatial Working Memory, and 4) Stop Signal Task. Trained study personnel will provide instructions and brief demonstrations according to the standard instructions for each module before the participant is tested.

Examination of decision-making is performed using The Iowa Gambling Task™ by Psychological Assessments Resources, Inc. The computer-based game is run using Inquisit 3 Lab software [19]. Trained study personnel read a scripted introductory statement, providing all of the required instructions. Participants are asked to complete the task on their own; study personnel step out of the testing room until the participant indicates that the task is complete.

### Eating behavior

Eating behaviors and motivations are assessed by questionnaire and food preference activities. The Yale Food Addiction Scale addresses addictive eating behaviors reported over the past 12 months, particularly with high-fat/sugar foods [10]. The Food Choice Questionnaire assesses nine motives related to food choice: 1) health, 2) mood, 3) convenience, 4) sensory appeal, 5) natural content, 6) price, 7) weight, 8) familiarity, and 9) ethical concern [12]. The Three-Factor Eating Questionnaire is used to measure: 1) ‘cognitive restraint of eating’, 2) ‘disinhibition’, and 3) ‘hunger’ [13].

Implicit and explicit wanting of foods is assessed using a paired foods test, which is based on the Implicit Associations Test [20]. A forced-choice paired comparison paradigm presents paired images of foods side-by-side on a computer screen. Participants are asked to select the food that they most want to eat by pressing either the L or D key on a standard keyboard, corresponding to the food image on the right or left of the screen, respectively. The food preference activity is administered only once, at approximately 1.5 h postprandial, with respect to the timing of consumption of the mixed macronutrient challenge meal. Study personnel provide instructions for the assessment, but are not present as it is being completed. This computerized task is designed to measure implicit liking and wanting of four classifications of foods: high-fat and sweet, high-fat and savory, low-fat and sweet, low-fat and savory. As part of this task, images of single food items are presented individually to determine explicit liking of particular foods. Participants are instructed to indicate how much they like the taste of a single food item by clicking a location along an electronic visual analog scale that is presented below the image of the single food item on the computer screen.

### MindWare mobile impedance cardiograph

Heart rate variability, respiration variability, and sympathetic nervous system activity as measured by skin conductance, is collected using the MindWare hardware and software systems by MindWare Technologies LTD. Data is monitored in real-time for the majority of one research visit day by wireless data transmission or captured locally on the collection device worn by the participant. Electrodes are placed in seven identified locations on the torso, five on the front torso and two on the back. Locations are predetermined by the manufacturer. Men are asked to shave the placement sites according to a provided diagram before the study visit. Trained study personnel clean skin at the application site using sterile alcohol prep pads and wipe dry using gauze before placing the torso electrodes. Two additional electrodes are placed on the non-dominant hand using only water to clean the hand locations. Data is acquired using the MindWare system for 15 min before and immediately after the two study challenges: 1) mixed macronutrient dietary challenge and 2) emotional recall challenge. Participants are asked to sit quietly during these dedicated data collection/rest periods without access to mobile devices or reading materials. Data is continuously collected throughout the morning and early afternoon, in addition to the described data collection/rest periods.

### Emotional challenge

The Profile of Mood States questionnaire is administered before and immediately after the anger recall task. Participants are asked to remember, relive, and vividly recall a negative event that occurred within the last six months that makes them feel extremely angry. A paper and pen are provided for taking notes and recording details of the memory for a period of 2 min. The participant is then asked to share the experience aloud with a study staff member for 3 min.

### Taste thresholds

Threshold concentrations of perceived taste of salty, sweet, and bitter flavors are assessed by a forced choice masked taste test. Solutions of varying concentrations of sodium chloride, sucrose, and caffeine, respectively, are prepared using distilled water at room temperature. Sensitivity to salty taste is assessed using five concentration levels: 1) 0.5 g/L, 2) 1.75 g/L, 3) 6 g/L, 4) 21 g/L, and 5) 75 g/L (approximately 10 times the concentration in chicken broth). Perception of sweet taste is assessed using five levels of sucrose concentration: 1) 1.23 g/L, 2) 3.7 g/L, 3) 11 g/L, 4) 33 g/L, and 5) 99 g/L (similar to the concentration found in regular soft drinks). Threshold of detection of bitter taste is assessed using five concentrations of caffeine in distilled water: 1) 0.16 g/L, 2) 0.25 g/L, 3) 0.40 g/L, 4) 0.64 g/L, and 5) 1.01 g/L (similar to the concentration in brewed coffee).

Solutions are presented to study participants in increasing order, starting with the most dilute and increasing in concentration until three consecutive concentrations are correctly identified. Samples are provided in clear, plastic cups labeled with randomly generated code numbers. The participant is instructed to rinse the mouth with distilled water before each round of tastings. Each round consists of three taste cups; one cup contains the tastant (solution) and two identical clear plastic cups that contain fresh distilled water only. The participant is asked to record the unique identifiers on each of the three cups presented at each round and then to select which solution is different from the other two. The participant records the three-digit number identifying the odd cup and if taste distinction is not possible, the participant is instructed to guess which solution is different. Participants may spit the taste solution out or swallow it, depending on preference, and are provided with more sample volume to taste, if requested. Sample cups are removed after each round and study personnel record the identifier of the selected cup behind a wall to determine if the taste solution was correctly identified. The lowest concentration correctly identified in a series of three consecutive correctly identified solutions is determined to be the threshold of detection.

### Skin reflectance

Skin reflectance is assessed using a Konica-Minolta Spectrophotometer CM-2500d as described previously [21]. The instrument is placed flat against the skin in three locations: 1) back of hand, in the area between the index finger and thumb, 2) inside of the upper arm, and 3) upper center of the forehead. Measurements are taken in duplicate and averaged.

### Endothelial function

Peripheral endothelial vasodilation responses to reactive hyperemia are assessed using an EndoPAT 2000 (endothelial peripheral arterial tone) [22] as a non-diagnostic indicator of endothelial function. The participant lies supine on a bed with legs uncrossed and must rest for 10–15 min. A blood pressure cuff is placed on the non-dominant upper arm and fit snuggly above the elbow. The wrists and forearms rest on arm supports positioned at a comfortable distance based on arm length. The index finger of each hand is placed in a probe that is positioned in a designated socket of the arm support. The probe covers inflate in order to ensure connectivity between the tip of the finger and the wire probe. A soft anchor is placed on the finger adjacent to the probe finger and the tubing is looped to avoid contact with the hand. The participant must lie still in order to produce a stable baseline period of data for at least 5 min. The upper arm blood pressure cuff is inflated to 200 mmHg and is kept at that pressure to occlude the brachial artery for 5 min. After 5 min, the blood pressure cuff is released and deflates quickly. The participant remains resting for 5 min post-occlusion. The reactive hyperemia index score is calculated as the post-to-pre occlusion PAT signal ratio on the occluded side, normalized to the control side and corrected for baseline vascular tone (Itamar Medical Ltd).

## Statistical analysis

### Sample size calculations

The sample size calculation is designed to detect a potentially small correlation between dietary variables and our primary outcomes, i.e. systemic immune activation and metabolomics responses to a mixed macronutrient challenge meal. In order to explain approximately 3% of the variation in inflammation, we need to detect a correlation coefficient (R) = 0.18 (R^2^ = 0.032 ≈ 3%). Establishing 95% power and α = 0.05 level of significance, the target sample size is 22 in each of the 18 categories defined by age, sex, and BMI. In addition, we aim to enroll an additional 10% of the calculated sample size to account for attrition.

### Data management

Study data is collected and managed using the Research Electronic Data Capture (REDCap™) tools hosted by the University of California Davis Health System Clinical and Translational Science Center [23]. REDCap is a secure, web-based application designed to support data capture for research studies, providing 1) an intuitive interface for validated data entry; 2) audit trails for tracking data manipulation and export procedures; 3) automated export procedures for seamless data downloads to common statistical packages; and 4) procedures for importing data from external sources.

Data quality is assured by several methods, including double data entry, electronic data checking, and laboratory-specific use of certified and non-certified controls and standards. Data that is collected in real-time during study visits is recorded in a master datasheet using MS Excel and directly entered into the REDCap™ database by the data manager. Using feature number 4 described above, the MS Excel sheet is periodically uploaded to the REDCap™ database, where inconsistencies between the manually entered data and the uploaded data are identified and flagged for investigation. As previously described, responses to questionnaires are entered directly into the database using the survey tool. Responses are downloaded from the REDCap™ database and entered into pre-programmed scoring sheets, which use the macros and conditional formatting capabilities of MS Excel. Surveys are scored according to published guidelines and scores are entered into the databases along with the original responses.

Statistical analysis of reliability and measurement error will be performed for assessment tools utilizing self-reporting methods, including the Profile of Mood States and Yale Food Addiction Scale questionnaires. Cronbach’s alpha will be calculated according to pre-defined dimensions for respective scales.

Biochemical data are generated according to assay manufacturer guidelines using certified controls whenever possible. Laboratory-specific use of standards and non-certified controls are recorded to evaluate data quality and maintain quality control.

### Anticipated results

Participant characteristics will be calculated as means and standard deviations, medians and percentiles, and percentages, overall and by phenotype, once determined. Differences in demographic variables and general characteristics across phenotypic groups will be tested using analysis of variance and logistic regression with multiple factors. Continuous variables will be mathematically transformed when data are not normally distributed, as needed. Non-parametric tests will be used for variables that do not meet assumptions for statistical tests after transformation attempts. Dietary quality scores will be calculated using standard methods including the Healthy Eating Index [24], which utilizes a density approach for specific intake per 1000 kcal or as a percent of caloric intake, or by cluster analysis of collected data. Recent and habitual macronutrient and micronutrient intake will be summarized from 24-h dietary recalls and food frequency questionnaires respectively and used in specific analyses. In the former, marked under- or over reporting will be identified using a ratio of energy intake: observed resting metabolic rate. In the latter, nutrient densities will be calculated and used in analyses.

Within each area of study, participants will be grouped using cluster analysis to create area-specific phenotypes. The four main areas of study are 1) Immunology and gut microbiota, 2) metabolomics, 3) dietary intake and physical activity, and 4) stress reactivity and cognitive function. Chi-square tests and loglinear models will be used to compare the defined phenotypes between areas of study.

Cluster analysis will be used to identify overall phenotypic groups within the study population. Component variables will be compared between clusters using analysis of variance. Sampling bins, overall phenotypic groups, and standard phenotypes will be compared using chi-square tests and loglinear models. Definitions of phenotypic groups from the literature will be applied to the current data set as “standard” phenotypic groups.

Metabolomics data will be used to calculate product: substrate ratios which will be explored as unique phenotypic descriptors [25]. Multi-omic analyses will employ partial correlations to estimate latent data networks [26]. Analysis-specific statistical plans will be published at a later date.

## Discussion

This study will provide insights into metabolic variability among healthy US adults that will help guide further research, including controlled intervention trials with foods or whole diets. Many aspects of immune function, metabolomics, physiology, neuroendocrinology, and lifestyle are being investigated in order to identify key factors that may differentiate metabolic health among individuals and thus be important covariates in future studies. The strengths of this study include the multiple dietary data collection tools used for assessment of dietary intake, the broad range of outcomes to be included in the phenotypic analysis, and a study design that minimizes losses to follow-up. The similarity of the challenge meal formula to one previously designed by European Nutrigenomics Organization [18] also provides the potential for comparison with phenotypic flexibility data in a US population to similar data collected in European populations. Limitations of this study include a lack of control group for comparison of the postprandial changes in response to the challenge meal and the unknown contamination of particular measurements that may result from performing other study tasks during the same day (e.g. effects of responding to a questionnaire about food choices on subjective feelings of appetite and hunger). With our study team’s broad range of expertise, we believe that the immense amount of biological data being collected during this study will reveal important differences in metabolic health and phenotypic flexibility.

## Abbreviations

BMI: Body mass index
REDCap: Research electronic data capture
VCO_2_: Volume carbon dioxide produced
VO_2_: Volume oxygen consumed
